# Ophiopogonin D′ inhibited tumour growth and metastasis of anaplastic thyroid cancer by modulating JUN/RGS4 signalling

**DOI:** 10.1111/jcmm.70014

**Published:** 2024-08-17

**Authors:** Tong Xu, Wanli Zhang, Yiwen Zhang, Feifeng Song, Ping Huang

**Affiliations:** ^1^ Center for Clinical Pharmacy, Cancer Center, Department of Pharmacy Zhejiang Provincial People's Hospital (Affiliated People's Hospital), Hangzhou Medical College Hangzhou Zhejiang China; ^2^ Key Laboratory of Endocrine Gland Diseases of Zhejiang Province Hangzhou Zhejiang China; ^3^ Institute of Pharmacology, Department of Pharmaceutical Sciences Zhejiang University of Technology Hangzhou Zhejiang China; ^4^ Clinical Research Center for Cancer of Zhejiang Province Hangzhou Zhejiang China

**Keywords:** anaplastic thyroid cancer, apoptosis, JUN, ophiopogonin D′, RGS4, transactivation

## Abstract

Anaplastic thyroid cancer (ATC), an aggressive malignancy with virtually 100% disease‐specific mortality, has long posed a formidable challenge in oncology due to its resistance to conventional treatments and the severe side effects associated with current regimens such as doxorubicin chemotherapy. Consequently, there was urgent need to identify novel candidate compounds that could provide innovative therapeutic strategies for ATC. Ophiopogonin D′ (OPD'), a triterpenoid saponin extracted, yet its roles in ATC has not been reported. Our data demonstrated that OPD' potently inhibited proliferation and metastasis of ATC cells, promoting cell cycle arrest and apoptosis. Remarkably, OPD' impeded growth and metastasis of ATC in vitro and in vivo, displaying an encouraging safety profile. Regulator of G‐protein signalling 4 (RGS4) expression was significantly up‐regulated in ATC compared to normal tissues, and this upregulation was suppressed by OPD' treatment. Mechanistically, we elucidated that the transcription factor JUN bound to the RGS4 promoter, driving its transactivation. However, OPD' interacted with JUN, attenuating its transcriptional activity and thereby disrupting RGS4 overexpression. In summary, our research revealed that OPD' bound with JUN, which in turn resulted in the suppression of transcriptional activation of RGS4, thereby eliciting cell cycle arrest and apoptosis in ATC cells. These findings could offer promise in the development of high‐quality candidate compounds for treatment in ATC.

## 1 INTRODUCTION

Thyroid cancer, a prevalent malignancy within the endocrine system, has witnessed a disconcerting rise in both incidence and mortality recently. According to the 2020 Global Cancer Observatory report, the global burden of thyroid cancer reached an alarming 586,000 cases.^1^ And the American Cancer Society recently announced that there were approximately 43,800 new cases of thyroid cancer in the United States (11,860 in men and 31,940 in women) and approximately 2230 deaths (1070 in men and 1160 in women) by 2022.^2^, ^3^, ^4^ Categorized based on cellular origin and differentiation, thyroid cancer encompasses several subtypes: papillary thyroid cancer, follicular thyroid cancer, medullary thyroid cancer, poorly differentiated thyroid cancer and anaplastic thyroid cancer (ATC).^5^ Despite comprising only 1%–2% of thyroid cancer cases, ATC stands out with its unparalleled malignancy,^6^, ^7^ accounting for a staggering 20%–40% of thyroid cancer‐related mortalities, with a near‐100% disease‐specific mortality rate.^8^, ^9^, ^10^ The average survival time of ATC patients was less than 6 months, which was drastically shorter than that of differentiated thyroid cancer. Traditional therapeutic methods and drugs, such as surgery, chemoradiotherapy and targeted therapy, have limited efficacy on ATC, which made the treatment of ATC patients in a long‐term dilemma and become a worldwide medical problem.^11^, ^12^, ^13^ Thus, there is an urgent demand for novel, efficacious and low‐toxicity molecules to surmount the existing therapeutic hurdles in ATC management.

Natural product‐derived small molecules have emerged as a cornerstone in the development of antitumor therapeutics, offering a rich reservoir for medicinal innovation.^14^, ^15^ Ophiopogonin, a class of steroid glycoside compounds extracted from the Liriope spicata (Ophiopogon japonicus), had been reported for exhibiting anti‐inflammatory and antioxidative properties, exerting functional roles in pathologies such as myocardial injury, obesity and osteoporosis.^16^, ^17^, ^18^, ^19^ Its antitumor potential has been confirmed across various cancer, including breast, prostate, lung and colorectal cancers, through the modulation of multiple signalling pathways critical for tumour initiation and progression.^20^, ^21^, ^22^, ^23^ Ophiopogonin D′ (OPD'), a triterpene saponin isolated from the extracts of Ophiopogon japonicus, remains understudied, particularly in relation to its antitumor activities and molecular mechanisms in ATC.

In this study, we have substantiated the anti‐ATC activities of OPD' by in vitro and in vivo experimental models. Moreover, we demonstrate that OPD' exerted its inhibitory effects on ATC initiation and metastasis by binding to JUN to suppress the transcriptional activation of Regulator of G‐protein signalling 4 (RGS4). Consequently, Our findings identified OPD' as a promising candidate for the development of targeted ATC therapies.

## 2 MATERIALS AND METHODS

### 2.1 Bioinformatics analysis

The datasets GSE76039,^24^ GSE33630^25^ and GSE29265 were retrieved from the Gene Expression Omnibus (GEO; https://www.ncbi.nlm.nih.gov/geo/). Subsequently, batch effects across these datasets were adjusted using the limma package in the R4.1.3 environment, thereby preserving inter‐group differences post‐normalization. In addition, the control group and 8505C cells treated with OPD' for 24 h were collected for transcriptome RNA‐seq (Oebiotech). Then differential expression analysis of genes was performed respectively for ATC compared to normal tissue (NT), OPD'‐treated group compared to control group using the limma package. The thresholds were set such that the *p*‐value <0.05 and | log_2_ (fold change) | >1. The Gene Ontology functional annotations were conducted for differentially expressed genes (DEGs) following OPD' treatment, alongside Gene Set Enrichment Analysis (GSEA) for the genes highly correlated with RGS4 expression, further elucidating the biological roles of DEGs among the distinct subgroups by the clusterProfiler package. And the Swiss Target Prediction database (http://www.swisstargetprediction.ch/) was applied to predict the transcription factors bound by OPD' possibly and Signalling Pathways Project database (http://www.signalingpathways.org/) to predict the transcription factors that might regulate RSG4. The molecular docking of JUN protein with candidate molecules was performed by using the MolAICal software.^26^

### 2.2 Cell culture

The normal thyroid cell line Nthy‐ori 3–1 (NTHY, Fenghui Biotechnology, SC2022010901), 293FT (Procell, CL‐0313) as well as ATC cell lines 8505C (Fenghui Biotechnology, CL0015), ARO (Zqxzbio, ZQ0686) and KHM5M (Procell, CL‐0623) were each cultured in Dulbecco's Modified Eagle Medium supplemented with 10% fetal bovine serum (FBS, NEWZERUM, FBSE500). These cell cultures were cultured in a humidified incubator with a controlled atmosphere at 37°C and 5% CO_2_.

### 2.3 siRNA transfection and lentiviral transduction

For siRNA transfection, ATC cells were plated into 6‐well culture plates at a confluence ranging from 35% to 45%, following which siRNA transfections were carried out according to the manufacturer's instructions using Transfection reagent (Abclonal, RM09014P). The target sequence for JUN siRNA #1 was CGGACCTTATGGCTACA‐GTAA. And for JUN siRNA #2, the target sequence was CGCAAACCTCAGCAAC‐TTCAA. To product recombinant lentivirus and stably expressing cell lines, Flag‐RGS4 expressing plasmid was co‐transfected with the packaging plasmid pRD8.9 and envelope plasmid pMD.G into 293FT cells to produce lentiviral particles. Upon successful cotransfection, polybrene at a concentration of 5 μg/mL was added to ATC cells cultured in 6‐well plates at a confluence of 35%–45%. Following a 48‐h incubation, the cells were subjected to selection with puromycin at 2.5 μg/mL to establish the stable cell line.

### 2.4 Cell proliferation, clone formation, scratch repair and transwell invasion

For cell proliferation assay, 2000 ATC cells were planted in 96‐well plates and treated with OPD' at 0, 1, 2, 3, 4 and 5 μM for 48 h after adhesion. The cell proliferation or viability was detected by CCK8 (FDbio, FD3788) or EdU (Beyotime, C0071L) according to the instructions. For clone formation assay, ATC cells were treated with OPD' at 0, 2 and 3 μM for 24 h. And 800 OPD'‐treated ATC cells were planted in 6‐well plates and cultured for 7 days. Then, it was stained with 0.1% crystal violet and photographed. For the scratch repair assay, the OPD'‐treated ATC cells were planted in a 12‐well plate, and a gap was evenly marked with the 200 μL tip after 100% fusion. The medium was changed to serum‐free medium and photographed at 0 and 24 h, respectively. For the transwell invasion assay, the upper chamber was first coated with 5% Matrigel (BD Biosciences, USA). 5*10^4^ OPD'‐treated ATC cells were inoculated with serum‐free medium in the upper chamber and then cultured with serum‐free medium in the lower chamber for 24 h. Then, it was fixed and stained with 0.1% crystal violet and photographed.

### 2.5 qRT‐PCR and dual‐luciferase reporter gene assay

The OPD'‐treated ATC cells were collected and used the RNA Extract Kit (AGbio, AG21023) to extract total RNA, which was then reversely transcribed into cDNA by the reverse transcription kit (AGbio, AG11706). The TBgreen qPCR Mix (Takara, RR820A) was used for PCR amplification according to the instructions. The forward primer of RGS4 was 5′‐TTCATCTCAGTCCAGGCAAC‐3′ and the reverse primer was 5′‐GGAATCCTTCTCCATCAGGTTG‐3′. The forward primer of JUN was 5′‐AGCC‐CAAACTAACCTCACG‐3′ and the reverse primer was 5′‐TGCTCTGTTTCAGGAT‐CTTGG‐3′. The forward primer of β‐actin was 5′‐CTGGAACGGTGAAGGTGACA‐3′ and the reverse primer was 5′‐AAGGAACTTCCTTGAACAATGCA‐3′. For dual‐luciferase reporter gene assay, ATC cells were collected after transfection and OPD'‐treated, and appropriate lysate was added. Then, the transcriptional activity of transcription factor was detected using the Dual Luciferase Reporter Gene Assay Kit (YEASEN, 11402ES60) according to the instructions.

### 2.6 Western blot

For western blot, ATC cells were treated with OPD' at 0, 2 and 3 μM for 48 h. And cell samples were harvested and lysed by RIPA lysis buffer (Applygen, C1053‐100). The ensuing protein extracts were quantified and denatured, after separated by 10% SDS‐PAGE gel electrophoresis, followed by electrotransfer onto PVDF membrane (Millipore, IPFL00010). Then, the PVDF membrane was blocked for 1 h at room temperature utilizing 5% BSA. The cleaved‐PARP (Abclonal, A19612), GAPDH (Proteintech, 10,494‐1‐AP), Cyclin D1 (Abclonal, A0310), RGS4 (Abclonal, A1787), β‐actin (Proteintech, 81,115‐1‐RR), p‐JUN (Abclonal, AP1190), or JUN (Abclonal, A0246) antibodies were utilized for the primary antibodies incubating overnight and the corresponding secondary antibodies were incubated at room temperature for 1 h and imaging.

### 2.7 Flow cytometry and immunohistochemistry

For apoptosis or cell cycle assay, ATC cells were treated with OPD' at 0, 2 and 3 μM for 48 h. And OPD'‐treated ATC cells were collected and used the Annexin V‐FITC/PI Apoptosis Kit (Liankebio, AP101C) or Cell Cycle Staining Kit (Liankebio, CCS012) according to the instructions to detect the apoptosis or cell cycle of ATC cells by flow cytometry (Beckman, USA). For immunohistochemistry, ATC and NT tissue samples were collected for detecting RGS4 expression. The tissue specimens were subsequently fixed, embedded in paraffin and sectioned. The resultant sections were deparaffinized and rehydrated sequentially using xylene and graded ethanol solutions. The slides were subjected to heat‐induced epitope retrieval in 1 mM EDTA at pH 8.0, and blocked with 5% goat serum to reduce nonspecific binding. The primary antibody was cultured with RGS4 (1:200) and the secondary antibody was biotinized for immunohistochemistry. For HE staining, paraffin‐embedded sections were deparaffinized in xylene and rehydrated through graded alcohols. Next, nuclei were stained with haematoxylin, followed by wash. Cytoplasm and extracellular matrix were then stained with eosin. After dehydration and clearing, the sections were mounted and examined under a microscope.

### 2.8 Animal models

8505C cells (3*10^6^) were re‐suspended in phosphate‐buffered saline (PBS) containing 10% Matrigel (v/v), and this suspension was then injected subcutaneously into nude mice at 3–4 weeks. When the tumour volume reached 100 mm^3^, they were randomly divided into two groups. PBS (for control group) or 2.5 mg/kg OPD' were intraperitoneally injected every 2–3 days, and body weight and tumour volume were measured. Tumour volume = length × width^2^/2. At the end, the mice were euthanized, and tumour tissue was collected and photographed. All animal tumour experiments were approved by Animal Ethics Committee of Zhejiang Provincial People's Hospital.

Zebrafish were maintained in a recirculating water system set at 28.5°C, characterized by seawater salinity of 200 mg/L sodium chloride, electrical conductivity ranging from 480 to 510 μS/cm, and pH maintained between 6.9 and 7.2. They were fed twice daily under a standardized 12‐h light–dark photoperiod with pelleted feed. For tumour growth and metastasis model, zebrafish embryos were manually dechorionated at 2 days post‐fertilization and anaesthetised with 0.15 mg/mL tricaine, and each groups of 10 zebrafish. ATC cells were labelled with 5 μg/mL DiI (Beyotime, C1036) at 37°C for 30 min, followed by two washes and resuspension in PBS. The labelled cells were then microinjected into the perivitelline space (500 cells/embryo, 10 μL) and incubated at 34°C. On Day 3 post‐injection, zebrafish were re‐anaesthetised, and fluorescent images were captured by fluorescence microscope. Fluorescence intensity and metastatic foci were quantified using Image Pro Plus software. The fluorescence density = (the optical density of fluorescence signal at Day 5)/(the optical density of fluorescence signal at Day 2). During the experiment, the number and mortality of toxic phenotypes such as live embryo, pericardial edema, cardiac stasis, cephalic and caudal necrosis, cephalic, and caudal malformation, cerebral haemorrhage and yolk sac edema were recorded under the microscope every 24 h.

### 2.9 Data analysis

All data were described as the mean ± standard deviation derived from three independent experiments. Statistical differences and significance between two groups were analysed using unpaired student's *t*‐tests implemented in GraphPad Prism version 7 software. Asterisks denoted statistical significance (*p < 0.05, **p < 0.01, ***p < 0.001).

## 3 RESULTS

### 3.1 OPD' inhibited the malignant phenotypes of ATC

**FIGURE 1:**
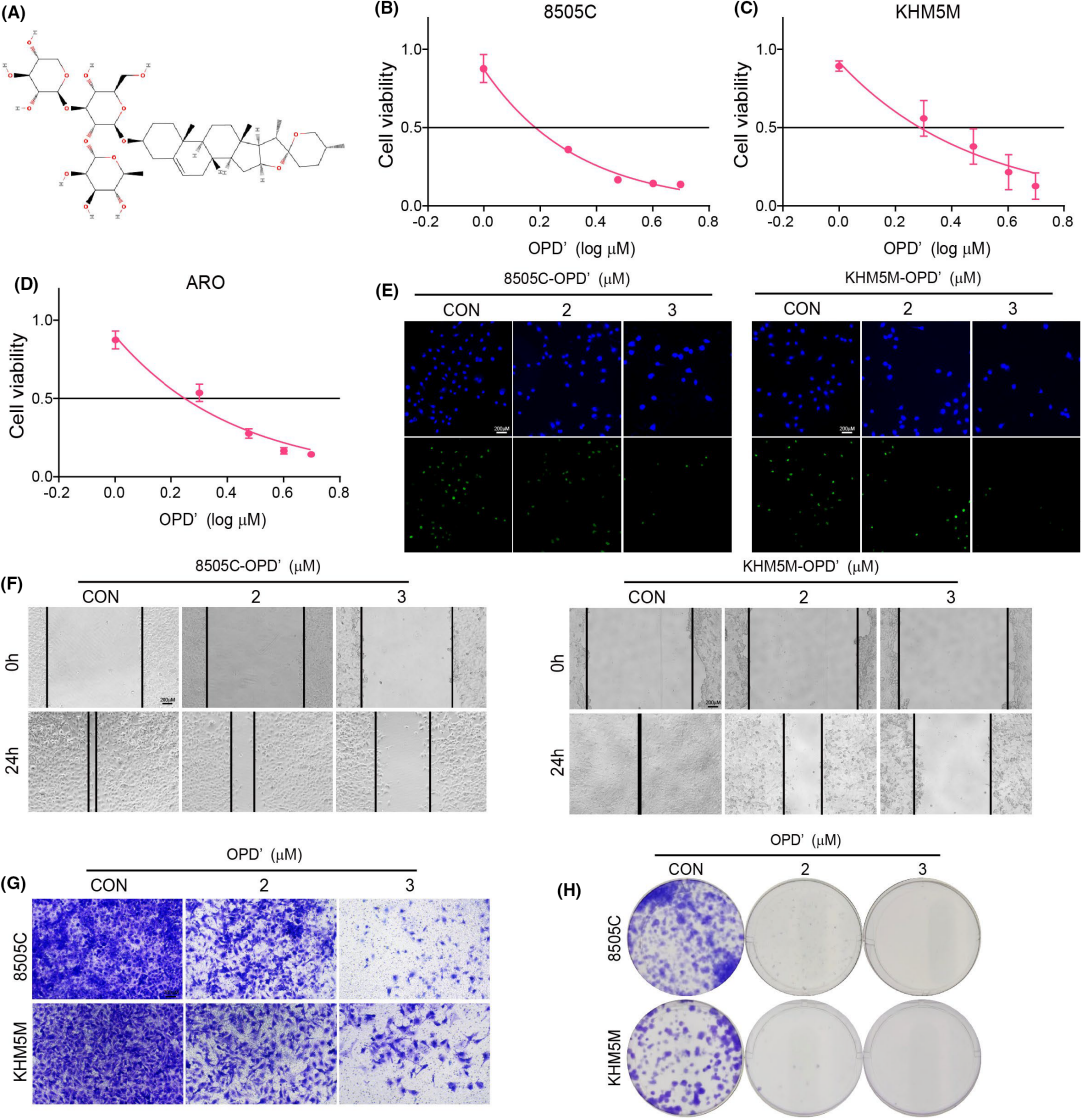
OPD' inhibited the malignant phenotypes of anaplastic thyroid cancers (ATC). (A) Chemical structure formula of OPD'. (B–D) The effects of different concentrations of OPD' on proliferation in ATC cell lines were detected by CCK8. (E) The effects of different concentrations of OPD' on proliferation in ATC cell lines were detected by EdU assay. (F) The effect of OPD' on migration ability of ATC cells was detected by scratch repair assay. (G) The effect of OPD' on the invasion ability of ATC cells was detected by Transwell assay. (H) The effect of OPD' on the clone formation ability of ATC cells was detected.

OPD' was a natural product derived from the Chinese medicine Ophiopogon japonicus, with a core platycodigenin structure (Figure 1A). Firstly, the effects of OPD' on the proliferation of ATC cell lines were examined (Figure 1B–D). The results showed that OPD' could significantly inhibit the growth of ATC cells 8505C, KHM5M and ARO, whose IC50 was 1.91, 2.34, and 2.16 μM, respectively. Further the 5‐ethynyl‐2′‐deoxyuridine (EdU) incorporation assay confirmed that a 24‐h exposure to OPD' markedly decelerated the proliferation of ATC cells (Figure 1E). Further, the scratch repair and invasion assay were used to determine the effect of OPD' on the metastasis ability of ATC (Figure 1F,G). Data results indicated that the migration ability of ATC cells was significantly decreased and the number of cells passing through the transwell chamber was reduced by OPD'. In addition, OP could also inhibit the clone formation ability of ATC cells (Figure 1H). Together, these results implied that OPD' obviously inhibited the malignant phenotypes of ATC cells.

### 3.2 OPD' induced cell cycle arrest and apoptosis in ATC

**FIGURE 2:**
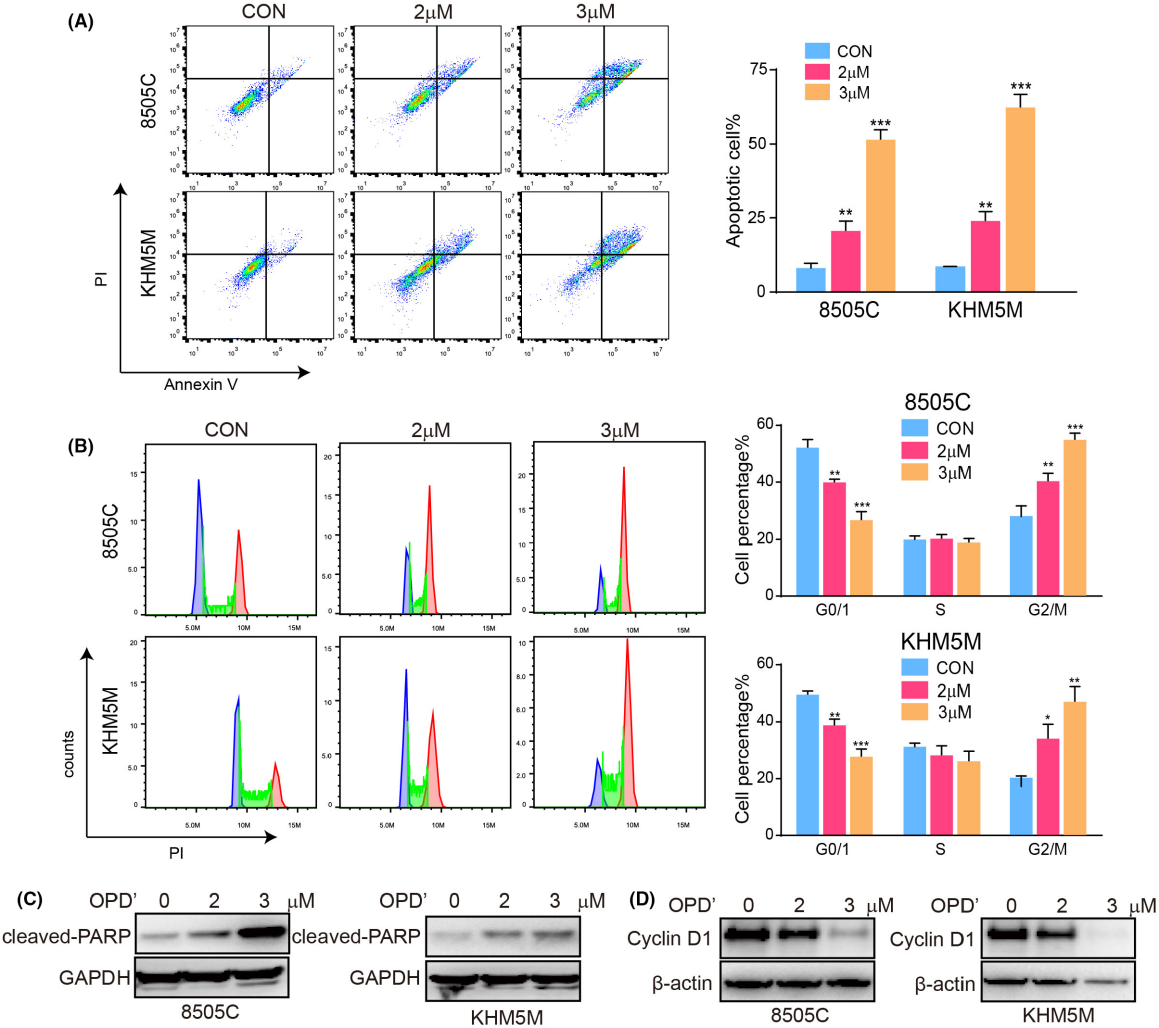
OPD' induced cell cycle arrest and apoptosis in anaplastic thyroid cancers (ATC). The effects of different concentrations of OPD' on (A) apoptosis and (B) cell cycle in ATC cell lines were detected by flow cytometry. The effects of different concentrations of OPD' on the expression of (C) cleaved‐PARP and (D) Cyclin D1 in ATC cell lines were detected by western blot.

Next, we tested the effect of OPD' treatment on apoptosis of ATC cells. Flow cytometry analysis revealed that upon treatment with 2 and 3 μM OPD', the percentage of apoptotic 8505C cells escalated to 20.71% and 51.73%, respectively, while the corresponding rate for KHM5M cells were 23.68% and 62.03% (Figure 2A). Then, the alterations in cell cycle distribution after OPD' treatment were investigated in the ATC. Data analysis suggested that OPD' could induce obvious cell cycle arrest in ATC cells, resulting in a decrease in G0/1 cells and increase in G2/M cells sharply (Figure 2B). Meanwhile, we observed the activation of apoptosis marker protein PARP and the expression level of cell cycle specific protein Cyclin D1 (Figure 2C,D). As expected, OPD' activated cleaved‐PARP and inhibited the expression of Cyclin D1. In conclusion, our results substantiate that OPD' exhibits a remarkable capacity to induce apoptosis and cell cycle arrest in ATC cells, providing a strong rationale for its further exploration as a therapeutic agent in the management of ATC.

### 3.3 OPD' suppressed the growth and metastasis of ATC in vivo

**FIGURE 3:**
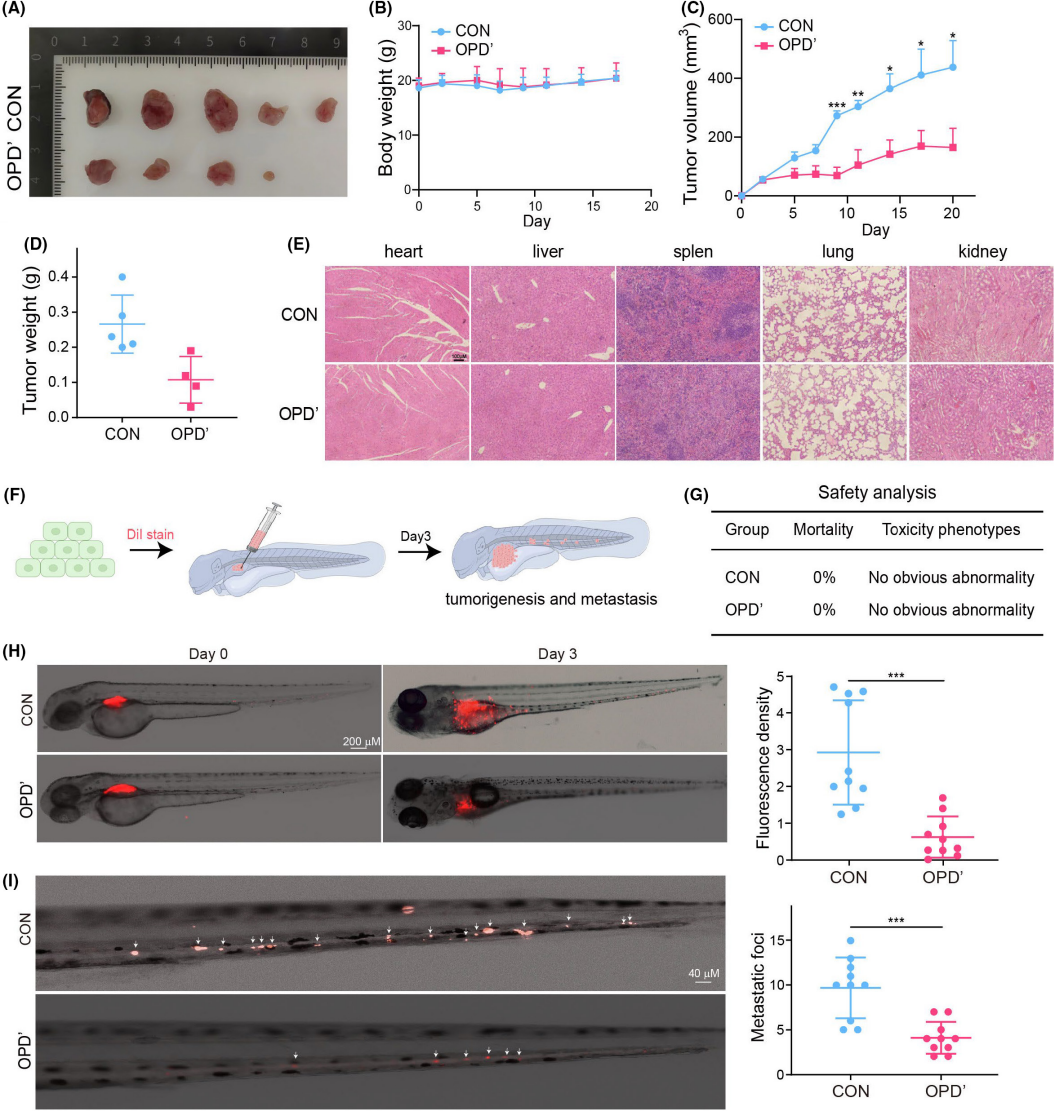
OPD' suppressed the growth and metastasis of anaplastic thyroid cancers (ATC) in vivo. (A) The effect of 2.5 mg/kg OPD' on ATC tumour growth was investigated in nude mice xenograft tumour model. (B) Mouse body weight. (C) Tumour growth curve. (D) Tumour weight. (E) The HE staining of heart, liver, spleen, lung and kidney of mice in control or OPD'–treated groups. (F) The effects of 2 μM OPD' on ATC tumour growth and metastasis were investigated in zebrafish xenograft tumour model. (G) Safety analysis of OPD'. (H) The fluorescence density of tumour in situ. (I) The number of tumour metastatic foci.

To substantiate the pharmacological activities of OPD' beyond in vitro assessments, we extended our investigations to in vivo models. Firstly, we constructed ATC xenografted tumour model in nude mice, followed by treatment with 2.5 mg/kg of OPD'. The data showed that the growth of ATC tumours was greatly inhibited after OPD' treatment and the endpoint weight of tumour tissues were significantly reduced, with no significant difference in body weight (Figure 3A–D). In addition, the pathological characteristics of the heart, liver, spleen, lung and kidney tissue of mice were not significantly changed by HE staining, suggesting its high security (Figure 3E). Meanwhile, zebrafish model had been applied to evaluate the effect of OPD' on ATC tumour metastasis due to its high efficiency and visualization advantages (Figure 3F). In vivo, OPD' treatment at 2 μM concentration was shown not to cause death and toxic phenotypes in zebrafish (Figure 3G). Moreover, after 3 days of tumour harbouring and drug administration, significant inhibition of tumour growth was observed (Figure 3H). The number of tumour metastatic foci in the zebrafish tail was greatly decreased after OPD' treatment (Figure 3I). In general, OPD' could remarkably impede the growth and metastasis of ATC tumours with highly effective and low toxic in vivo.

### 3.4 RGS4 may be the crucial protein in the anti‐ATC activity of OPD'

**FIGURE 4:**
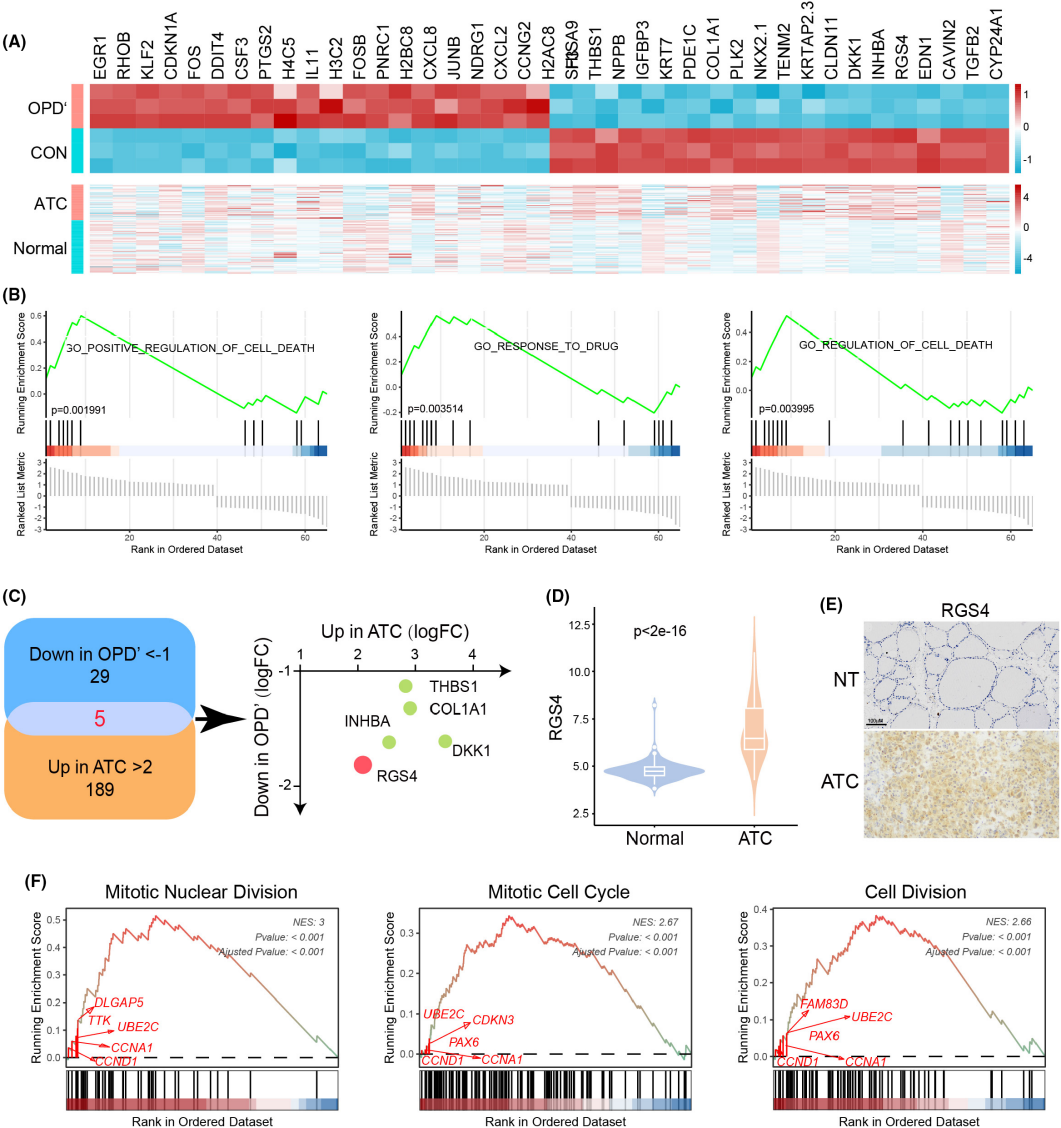
Regulator of G‐protein signalling 4 (RGS4) may be the crucial protein in the anti‐anaplastic thyroid cancers (ATC) activity of OPD'. (A) The heatmap of top20 differentially expressed genes (DEGs) and their expression in ATC tissues after OPD' treatment. (B) The Gene Set Enrichment Analysis (GSEA) analysis of DEGs. (C) The intersection of genes whose down‐regulation was less than −1 after OPD' treatment and genes whose up‐regulation was more than 2 in ATC. RSG4 expression in (D) ATC expression dataset and (E) IHC staining of clinical samples. (F) The GSEA enrichment analysis of RSG4 high expression related genes.

To elucidate the molecular underpinnings of OPD'‐mediated growth inhibition in ATC cells, we undertook a comprehensive transcriptomic analysis comparing untreated controls with OPD'‐treated ATC cells. The heatmap depicted the expression patterns of the top 20 upregulated and downregulated genes following OPD' treatment, showing their differential expression in ATC tissues (Figure 4A). GSEA analysis indicated that differential expressed genes were mainly enriched in positive regulation of cell death, response to drug and regulation of cell death signalling pathway (Figure 4B). Further five genes (RGS4, INHBA, DKK1, COL1A1 and THBS1) that were significantly up‐regulated in ATC tissue and down‐regulated after OPD' treatment were screened (Figure 4C). Among them, the expression of RGS4 changed the most, and its high expression in ATC was verified by experiments (Figure 4D,E). In addition, we analysed the pivotal signalling pathways regulated by RGS4 in ATC based on its expression (Figure 4F). The results showed that RGS4 was highly likely to mediate mitotic nuclear division, mitotic cell cycle and cell division signals through regulating CCND1, CCNA1 and UBE2C. In short, RGS4 might act crucial roles in OPD' inhibiting ATC.

### 3.5 RGS4 was necessary for OPD'‐induced cell cycle arrest and apoptosis in ATC

**FIGURE 5:**
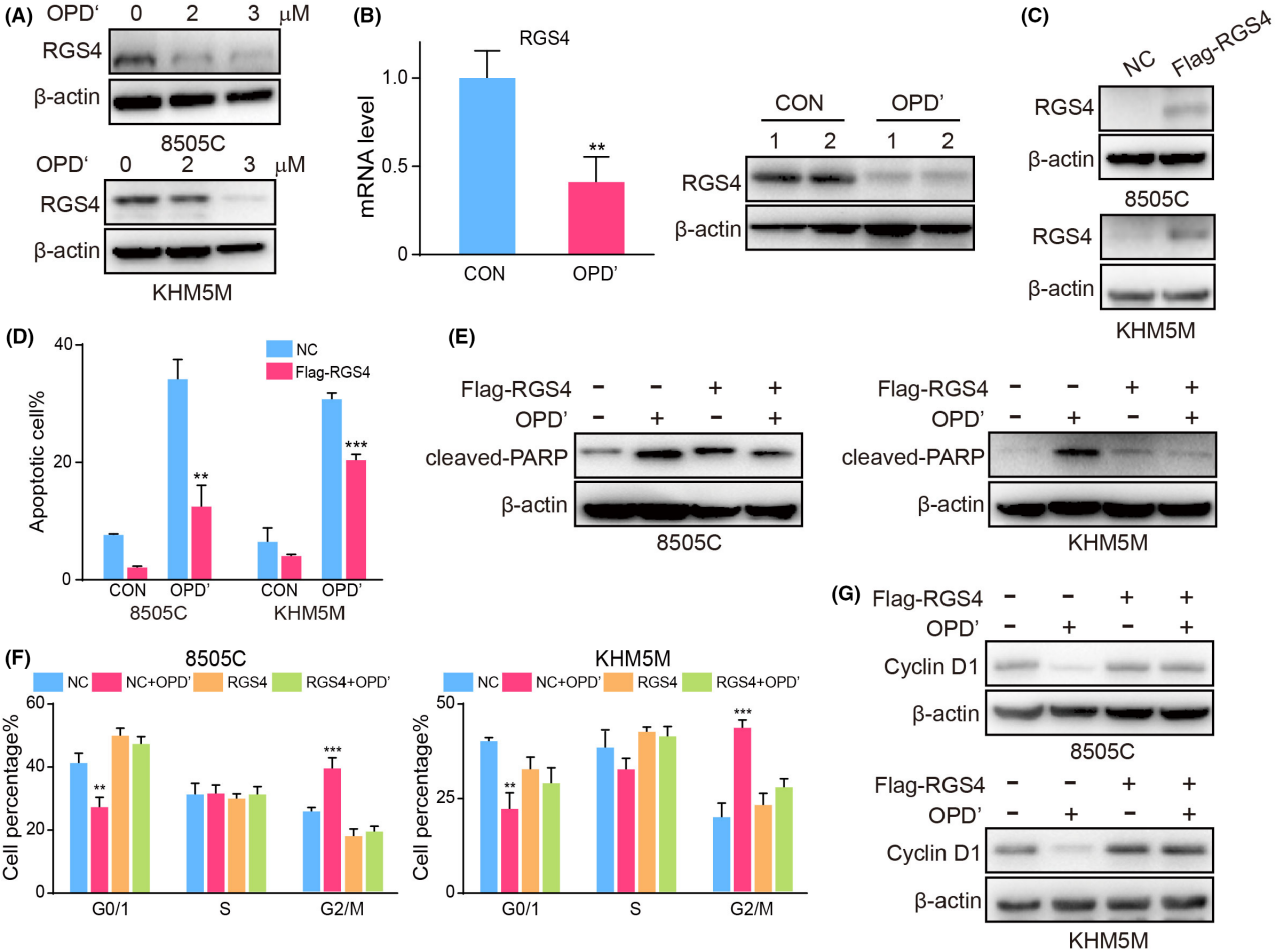
Regulator of G‐protein signalling 4 (RGS4) was necessary for OPD'‐induced cell cycle arrest and apoptosis in anaplastic thyroid cancers (ATC). (A) The effects of different concentrations of OPD' on the expression of RSG4 in ATC cell lines were detected by western blot. (B) The mRNA and protein expression of RGS4 in endpoint tumour tissues of control and OPD'‐treated groups. (C) The overexpression efficiency of RSG4 in ATC cell lines were detected by western blot. (D) The effects of OPD' on apoptosis in NC or RGS4 overexpressed ATC cell lines were detected by flow cytometry. (E) The effects of OPD' on the expression of cleaved‐PARP in NC or RGS4 overexpressed ATC cell lines were detected by western blot. (F) The effects of OPD' on cell cycle in NC or RGS4 overexpressed ATC cell lines were detected by flow cytometry. (G) The effects of OPD' on the expression of Cyclin D1 in NC or RGS4 overexpressed ATC cell lines were detected by western blot.

Given that the potential significant functions of RGS4 in the anti‐tumour activities of OPD', we first verified the effect of OPD' on RGS4 expression. Indeed, OPD' could effectively supress the protein level of RGS4 (Figure 5A). And a similar trend has been confirmed in animal models (Figure 5B). We then constructed ATC cell lines with stable overexpression of RGS4 to explore the effects of RSG4 on the anti‐tumour activities of OPD' (Figure 5C). Subsequently, flow cytometry indicated that overexpression of RGS4 could repair OPD'‐induced apoptosis of ATC cells and inhibit the expression of activated cleaved‐PARP (Figure 5D,E). In cycle arrest, the proportion of G0/1 cells down‐regulated by OPD' was recovered, and there was no significant difference in the proportion of G2/M cells up‐regulated by OPD', and the reduced the expression of Cyclin D1 was repaired (Figure 5F,G). Taken together, RGS4 was necessary for OPD'‐induced cell cycle arrest to promote ATC apoptosis.

### 3.6 OPD' directly bound JUN to transcriptionally regulate RGS4 expression

**FIGURE 6:**
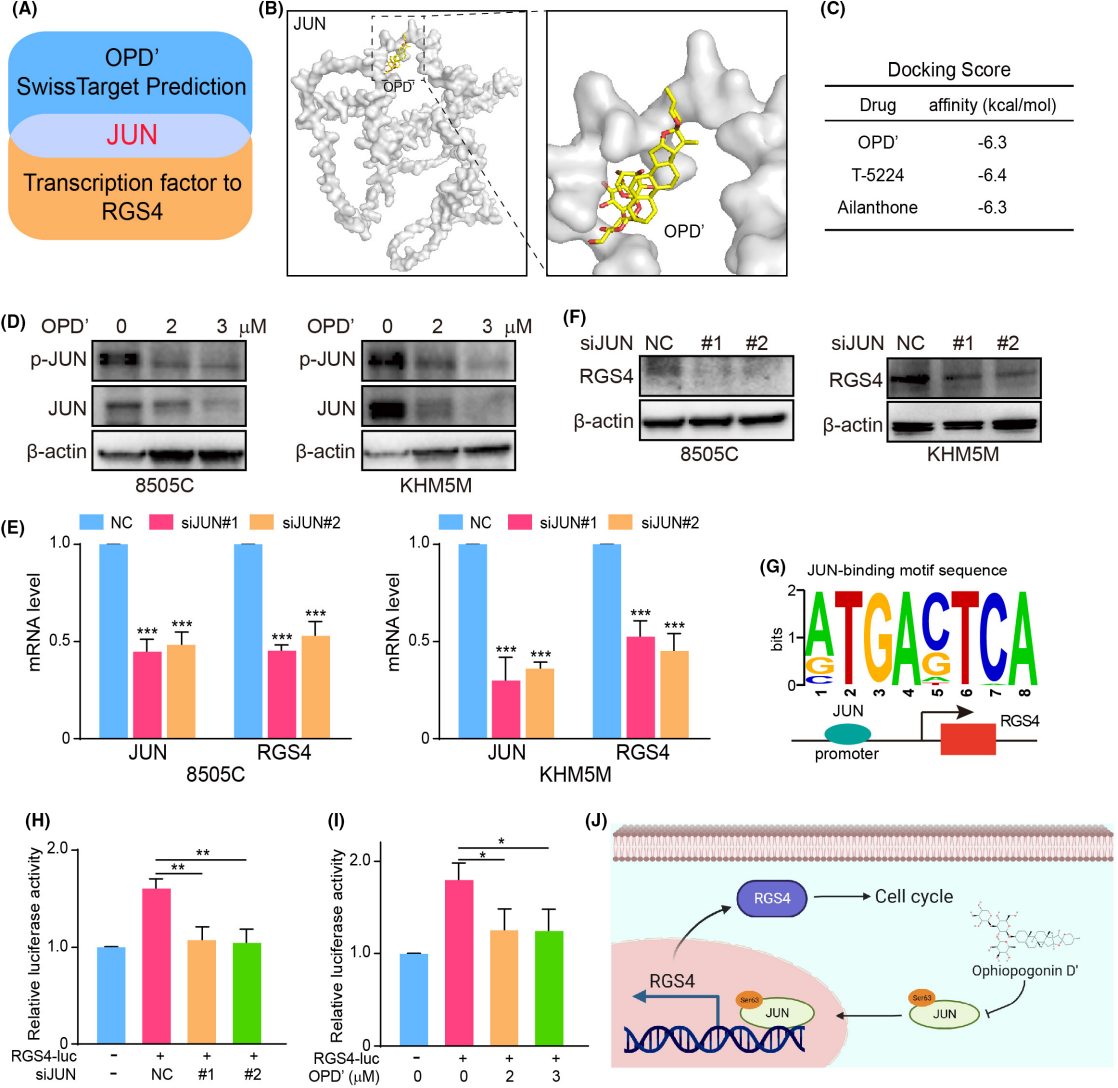
OPD' directly bound JUN to transcriptionally regulate Regulator of G‐protein signalling 4 (RGS4) expression. (A) The intersection of genes potentially whose was predicted to bind OPD' by Swiss Target Prediction database and genes whose transcriptionally regulate the expression of RGS4 by Signalling Pathways Project database. (B) The molecular docking of JUN with OPD'. (C) The docking score of OPD', T‐5224 and Ailanthone with JUN. (D) The effects of different concentrations of OPD' on the expression of p‐JUN and JUN in ATC cell lines were detected by western blot. (E) The effects of JUN silence on the expression of RGS4 in ATC cell lines were detected by qPCR. (F) The effects of NC or siJUN on the expression of RGS4 in ATC cell lines were detected by western blot. (G) The binding motif of JUN in upstream sequence of RGS4 promoter in JASPAR database. (H) The effect of silence of JUN on RGS4 transcriptional activation by Dual‐luciferase assay. (I) The effect of OPD' on RGS4 transcriptional activation by Dual‐luciferase assay. (J) The schematic diagram of the mechanism of OPD' anti‐tumour activity.

Given the marked reduction in RGS4 expression following OPD' treatment, we embarked on identifying potential transcription factors that could be influenced by OPD' to modulate RGS4. The Swiss Target Prediction database was applied to predict the transcription factors bound by OPD' possibly and Signalling Pathways Project database to predict the transcription factors that might regulate RSG4, and the crucial candidate transcription factor JUN was found by the intersection of the above genes (Figure 6A). To validate the potential interaction between OPD' and JUN, we employed molecular docking assay. The results showed that OPD' could bind JUN directly, and the docking score was similar to that of JUN inhibitors T‐5224 and Ailanthone that had been reported^27^, ^28^ (Figure 6B,C). Further analysis revealed that OPD' significantly repressed the phosphorylation and total protein levels of JUN (Figure 6D). Moreover, the experiments verified that silence of JUN could greatly reduce expression of RGS4 in mRNA and protein levels (Figure 6E,F). In addition, we had found the binding motif of JUN in upstream sequence of RGS4 promoter in JASPAR database (Figure 6G). Therefore, we added the upstream sequence of RGS4 promoter to the Dual luciferase reporter gene system plasmid to detect the transcriptional regulation of RGS4 by JUN. The results exhibited that both silence of JUN and OPD' treatment could significantly decrease the fluorescence signal, suggesting the transcriptional activation of RGS4 by JUN (Figure 6H,I). In general, OPD' directly bound JUN to transcriptionally regulate RGS4 expression.

## 4 DISCUSSION

Despite its relatively low prevalence within the thyroid cancer spectrum and among all malignancies, it was characterized by rapid pathological progression and carried the poorest prognosis among thyroid cancers.^29^ Diagnosed predominantly at advanced stages, ATC evades curative interventions due to its narrow therapeutic window.^30^ The standard clinical treatment regimen for ATC involved the combination of doxorubicin chemotherapy and radiotherapy, primarily aimed at triggering the apoptosis of tumour cells to impede disease progression.^31^ While this strategy could be efficacious, it was associated with significant side effects and prone to the development of drug resistance. Notwithstanding the FDA approval of the combination therapy of dabrafenib and trametinib for treating ATC patients harbouring BRAF V600E mutation, only approximately 40% of ATC cases exhibited such mutation. Moreover, the recent exploration of immunotherapy with the PD‐1 inhibitor nivolumab has failed to significantly impact overall survival in ATC patients, underscoring the ongoing challenge in addressing this malignancy.^32^ Consequently, there was an urgent need to identify novel, highly efficacious and less toxic small molecules that could provide innovative strategies for the clinical management of ATC. Here, we had ascertained through both in vitro and in vivo assay that OPD' could effectively inhibit the proliferation and metastasis of ATC, while exhibiting a favourable safety profile. Building upon these findings, the structural modification and enhancement of the bioactivity of OPD' may hold promise for the development of superior candidate compounds for therapeutic intervention against ATC.

Aberrant activation of mitotic and cell cycle signalling pathways is a hallmark of ATC, where deregulated expression of key functional proteins, such as CDKN2A, CDC25C, and Cyclin D1, accelerates its aggressive course.^33^, ^34^, ^35^ Prior studies have underscored the therapeutic potential of targeting these proteins to inhibit ATC cell proliferation and migration. Our investigation reveals that OPD' exerts potent antitumor effects in ATC by inducing cell cycle arrest and triggering apoptosis. Moreover, the expression of RGS4 was remarkably down‐regulated after treatment with OPD'. RGS4, a member of the RGS protein family, modulated G protein‐coupled signal transduction by influencing the activity of guanosine triphosphatase‐activating proteins (GAPs). As a negative regulator of G protein‐coupled receptors, RGS4 engages in complex signalling interactions with receptors, effectors and scaffold proteins, thereby influencing the localization, activity and stability of intracellular signals.^36^ Moreover, RGS4 had been implicated in the enhancement of cell viability or invasiveness in lung cancer, gliomas, ovarian cancer, colorectal cancer and breast cancer.^37^, ^38^ However, the roles of RGS4 in ATC remained relatively unexplored. Our study revealed a significantly elevated expression of RGS4 in ATC, with high RGS4 levels closely linked to dysregulated mitotic and cell cycle signalling pathways, thus implicating its pivotal roles in the malignant progression of ATC.

Further mechanistic investigations suggested that OPD' may interact with the transcription factor JUN, thereby attenuating its transcriptional activity. Importantly, JUN was identified as the transcriptional regulator of RGS4, and suppression of JUN effectively diminished the transcription of RGS4. We first used bioinformatics tools to predict the presence of JUN binding sites in the promoter region of RGS4, that is, the potential to receive JUN transcriptional regulation. Based on this, we confirmed the direct transcriptional activation of RGS4 by JUN using the Dual‐Luciferase Reporter Gene System. The JUN‐RGS4 signal axis exerts a critical force in its anti‐tumour effect. It is undeniable that there are other possible pathways or interactions that could also play significant roles. Deng et al. found that miR‐3663‐3p could regulate the expression of RGS4.^39^ Ghil et al. found that co‐expression of PAR4 and Gαq induced subcellular localization of RGS4 from the cytoplasm to the plasma membrane.^40^ Wick et al. found that CCI‐779 regulated the expression of RGS4 by inhibiting mTOR signals.^41^ However, whether these potential regulators of RGS4 were involved in the anti‐ATC activity of OPD' remained unclear and required further investigation and experimental verification.

This significant effect of OPD' indicated that the natural compound may have a narrow therapeutic window, underscoring the importance of precise drug delivery. Our study initially confirmed the safety of OPD' in zebrafish models and mouse models, and we will further expand our research. ADME in‐depth studies are conducted to understand the compound's behaviour in vivo and explore its high affinity and sensitivity to targets. A more comprehensive toxicity study is performed to determine a safe concentration range for the compound without side effects. We are also aware that individual differences may affect drug metabolism and response, and therefore plan to develop a personalized dosing regimen by closely monitoring blood concentrations and patient response in future clinical trials. Through these expanded studies, we will be able to more fully understand the therapeutic potential and limitations of OPD', while also providing a solid scientific basis for future clinical applications.

## 5 CONCLUSION

Taken together, our study robustly substantiated the promising anti‐tumour activity of OPD' in ATC and elucidated the mechanism that OPD' bind with JUN to repress RGS4 transcription, consequently inducing cell cycle arrest and apoptosis in ATC cells. These results could provide potential small molecule for clinical therapeutic strategy of ATC. These results could provide potential small molecule for clinical therapeutic strategy of ATC, and further exploration was needed for clinical transformation and therapeutic administration.

## AUTHOR CONTRIBUTIONS

**Tong Xu:** Project administration (equal); resources (equal). **Wanli Zhang:** Data curation (equal); validation (equal); visualization (equal). **Yiwen Zhang:** Supervision (equal); validation (equal). **Feifeng Song:** Methodology (equal); writing – original draft (equal). **Ping Huang:** Resources (equal); software (equal).

## FUNDING INFORMATION

The research was funded by the Chinese Medicine Research Program of Zhejiang Province (No. 2024ZL275 and 2024ZL242); National Natural Science Foundation of China (No. 82203858 and 82,161,138,019); Zhejiang Province Triangle health research Foundation (No. 2023CSJ‐3‐1002).

## CONFLICT OF INTEREST STATEMENT

All authors declared no competing interests.

## Data Availability

Data will be available on request.
